# Evaluating Emotional Well-Being after a Short-Term Traditional Yoga Practice Approach in Yoga Practitioners with an Existing Western-Type Yoga Practice

**DOI:** 10.1155/2016/7216982

**Published:** 2016-03-30

**Authors:** Maxi Meissner, Marja H. Cantell, Ronald Steiner, Xavier Sanchez

**Affiliations:** ^1^Department of Organizational Psychology, Faculty of Behavioural and Social Sciences, University of Groningen, Netherlands; ^2^Centre for Special Educational Needs and Youth Care, Faculty of Behavioural and Social Sciences, University of Groningen, Netherlands; ^3^Sport- und Rehamedizin Universitätsklinikum Ulm, Germany; ^4^Department of Medical and Sport Sciences, University of Cumbria, Lancaster, UK

## Abstract

The purpose of the present study was to examine the influence of a traditional yoga practice approach (morning daily practice, TY) compared to that of a Western yoga practice approach (once-twice weekly, evening practice, WY) on determinants of emotional well-being. To that end, in a pre/posttest between-subject design, measures of positive (PA) and negative affect (NA), mindfulness, perceived stress, and arousal states were taken in 24 healthy participants (20 women; mean age: 30.5, SD = 8.1 years) with an already existing WY practice, who either maintained WY or underwent a 2-week, five-times-per-week morning practice (TY). While WY participants maintained baseline values for all measures taken, TY participants showed significant beneficial changes for PA, NA, and mindfulness and a trend for improved ability to cope with stress at the completion of the intervention. Furthermore, TY participants displayed decreased subjective energy and energetic arousal. Altogether, findings indicate that the 2-week TY is beneficial over WY for improving perceived emotional well-being. The present findings (1) undermine and inspire a careful consideration and utilization of yoga practice approach to elicit the best benefits for emotional well-being and (2) support yoga as an evidence-based practice among healthy yoga practitioners.

## 1. Introduction

Over the twentieth century yoga has become a popular practice in the Western world, with an estimated 30 million people regularly practicing yoga worldwide [1]. The practice of yoga consists of eight limbs (universal moral and ethical principles, individual self-restraint, physical postures, breath control, calming of the senses, concentration, meditation, and pure contemplation) and is thought to facilitate health and well-being [2]. In fact, as substantiated by an ever-growing body of empirical research, yoga is emerging as a complementary practice improving physical, mental, and, especially, emotional health [3].

Parameters associated with emotional health entail affect, mindfulness, perceived stress, and arousal states. Affect is the automatic evaluation of a stimulus as positive (positive affect, PA) or negative (negative affect, NA). While NA is associated with increased subjective stress, aversive mood states, anxiety, depression, low self-esteem, and narrow attention [4, 5], PA is associated with optimism and greater tendencies to cope [6]. Mindfulness has been described as the systematic development of the ability to nonjudgmentally direct attention towards events in the field of consciousness [7]. Perceived stress represents the degree to which one's life is appraised as stressful [8] and furthermore, increased levels of perceived stress are associated with increased emotional distress [9]. Generally speaking, any state of arousal is located on a particular level of two bipolar dimensions, that is, energetic (EA) and tense arousal (TA). EA ranges from tiredness at the low end to energy at the upper end while TA ranges from calmness at the low end to tension at the upper end [10]. Arousal states have been linked with valence (pleasure versus displeasure); in other words, high energetic arousal (energy) and low tense arousal (calmness) are perceived as pleasant or positive. In contrast, high tense arousal (tension) and low energy (tiredness) are perceived as unpleasant or negative [11].

Altogether, affect, mindfulness, perceived stress, and arousal states have all been demonstrated to change into a beneficial direction when exposing previously yoga-naïve subjects, healthy [12–16] as well as clinical populations [11, 17], to yoga. Notably, most previous research concerning the effect of yoga on parameters of emotional well-being has largely concentrated on previously yoga-naïve participants. The effectiveness of an existing yoga practice on emotional well-being has not yet received much attention.

With regard to yoga practice frequency, previous yoga intervention studies have mostly focused on three different types of yoga practitioners: 1–3-times-weekly practice [18], >3-times-weekly practice [19], or previously yoga-naïve subjects exposed to 1–3-times-weekly yoga practice [11, 13, 14]. These more recreational practice frequencies are in strong disparity to how yoga is traditionally practiced, that is, a daily practice in the morning. This type of frequent practice is advocated to facilitate psychological and physiological health most effectively [20].

Notably, in a cross-sectional study of American Iyengar practitioners average practice frequencies (class attendance combined with home practice) amounted to about 4-5-times-weekly yoga practice [21], paralleling traditional practice frequencies. In this survey, class practice frequency was found an independent predictor of subjective well-being and mindfulness and negatively related to sleep disturbance [21]. Importantly, per day of additional home practice benefits were further increased for subjective well-being and sleep quality. In this cross-sectional survey average practice frequencies (class attendance combined with home practice) amounted to about 4-5-times-weekly yoga practice, altogether providing support for the advocated traditional practice approach. Yet, taking into account the cross-sectional and anonymous survey nature of the previous study, causality cannot be implied. Moreover authors reported a dropout rate of 27% which may have biased the results.

Thus, although cross-sectional data suggests that the traditional way of practicing yoga provides further health outcome benefits compared to the Western practice approach, yoga practice frequency has so far not been directly manipulated in a yoga intervention study. Therefore, the effects of an ongoing and more traditional daily practice have not yet been established.

The aim of the current study was to evaluate the effectiveness of a short-term (two-week) traditional-type yoga practice (five mornings per week, TY) with an ongoing once-to-twice-per-week evening yoga practice (Western yoga approach, WY) on parameters that measure perceived emotional health. More specifically, we compared affect scores using PA and NA (as measured by the Positive Affect and Negative Affect Schedule, PANAS [22]), mindfulness scores (the Mindful Attention Awareness Scale, MAAS [23]), perceived stress levels (the Perceived Stress Scale, PSS [8]), and arousal states such as energy, tension, tiredness, and calmness (the Activation-Deactivation Adjective Checklist, AD ACL [10]) before and at the end of a 2-week TY intervention. Findings from the current study will in their part help to better understand the psychological health outcomes and effectiveness related to Western and traditional approaches to practicing yoga. Ultimately, results of this study promote specific yoga practice prescriptions to better integrate yoga as an evidence-based alternative and complementary practice for emotional health benefits.

## 2. Materials and Methods

### 2.1. Participants

Twenty-four healthy subjects (20 women; mean age: 30.5, SD = 8.1 years) living in the northern part of Netherlands who were already practicing ashtanga yoga once or twice per week for a minimum of at least three months were recruited from a yoga school. Based on personal preference and time availability for the following two weeks, participants chose (convenience sample) to either maintain their regular yoga practice of once or twice per week in the evening time (Western yoga practice approach, WY, *n* = 12) or engage in a yoga practice at a frequency of five times per week between 07:00 h and 08:30 h in the morning (traditional approach to yoga practice, TY, *n* = 12).

### 2.2. Instruments

The following validated and widely used measures were administered at baseline and at the end of the two-week TY intervention (2-week point): the Positive Affect and Negative Affect Schedule (PANAS) [22], the Mindful Attention Awareness Scale (MAAS [23]), the Perceived Stress Scale (PSS) [8], and the Activation-Deactivation Adjective Checklist (AD ACL) [10].

The PANAS [22] was used to assess participants' mood over the previous week. It comprises 20 items and two subscales of 10 items each: positive affect (PA, score range 1–5; higher values correspond to more positive affect) and negative affect (NA, score range 1–5; higher values correspond to more distress). Cronbach's *α* was 0.83 and 0.78 for PA, at baseline and the 2-week point, respectively, and 0.88 and 0.88 for NA at baseline and the 2-week point, respectively. The Mindful Attention Awareness Scale (MAAS) [23] assessed participants' core characteristics of dispositional mindfulness over the previous week, specifically, receptive awareness of and attention to what is taking place in the present. It contains 15 items on a scale called mindfulness. Scores range from 1 to 6 and higher scores correspond to more mindfulness. Cronbach's *α* was 0.85 at baseline and *α* = 0.87 at the 2-week point. Perception of stress was determined by the Perceived Stress Scale [8]. The PSS contains 10 items that assess participants' feelings and thoughts during the previous week and produce one scale: perceived stress. Cronbach's *α* was 0.87 at baseline and 0.85 at the 2-week point. To evaluate arousal states over the previous week, the 20-item Activation-Deactivation Adjective Checklist (AD ACL) was used [24]. The AD ACL consists of four subscales (5 items each): energy (score range 1–4; higher scores correspond to more energy; Cronbach's *α* was 0.81 at baseline and 0.86 at the 2-week point), tiredness (score range 1–4; higher scores correspond to more tiredness; Cronbach's *α* was 0.86 at baseline and 0.88 at the 2-week point), tension (score range 1–4; higher scores correspond to more tension; Cronbach's *α* was 0.82 at baseline and 0.73 at the 2-week point), and calmness (score range 1–4; higher scores correspond to more calmness; Cronbach's *α* was 0.70 at baseline and 0.60 at the 2-week point). Combining the subscales energy and tiredness yields energetic arousal (EA) while combining the subscales tension and calmness yields tense arousal (TA).

Moreover, the Measure of Affect Regulation Style (MARS) [25], trait questionnaire, was used to measure participants affect regulation patterns at baseline. The 38 items assess how frequently participants utilize certain affect regulatory strategies. It consists of seven subscales, each scoring from 1 to 7 (higher scores corresponding to increased engagement in each strategy): active distraction (Cronbach's *α* was 0.73 at baseline), cognitive engagement (Cronbach's *α* was 0.77), behavioral engagement (Cronbach's *α* was 0.51), venting and expressing affect (Cronbach's *α* was 0.74), passive distraction (Cronbach's *α* was 0.11), rumination and withdrawal (Cronbach's *α* was 0.43), and waiting and reframing (Cronbach's *α* was 0.37). It is worth noting that the last three subscales yielded low internal consistency, which appears to be similar to prior work using the MARS [25]. Despite this, all subscales were included for analyses (specially for the passive distraction subscale). A demographic questionnaire was completed by each participant including age, gender, and the number of years/months of yoga practice.

Lastly, a manipulation check was undertaken during the first and the second week of the TY intervention in order to ensure that the perception of the yoga classes and the teacher's performance was similar in both groups. Immediately after each yoga class, participants rated the quality of each yoga class and the performance of the teacher by means of a visual-analogue scale—marking on a 10 cm line where the point at 0 cm corresponded to “very poor” and at 10 cm corresponded to “outstanding.”

### 2.3. Procedure

**Figure 1:**
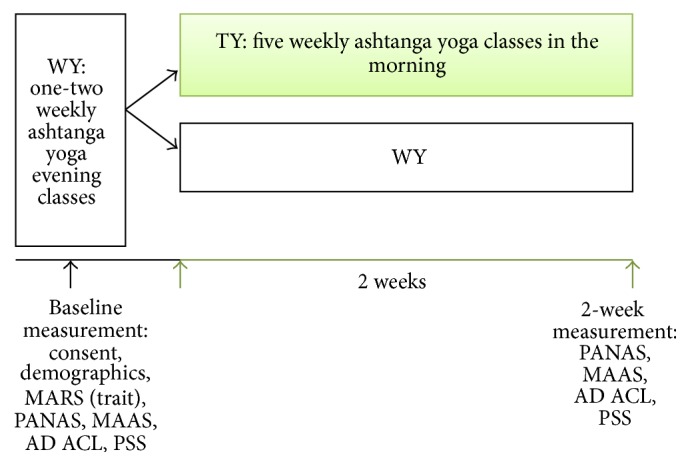
Research design. WY = Western yoga practice approach (yoga practice one to two times per week, in the evening), TY = traditional yoga practice approach (yoga practice five times per week in the morning), MARS = Measure of Affect Regulatory Style, PANAS = Positive Affect and Negative Affect Schedule, MAAS = Mindful Attention Awareness Scale, AD ACL = Activation-Deactivation Adjective Checklist, and PSS = Perceived Stress Scale.

A pre/posttest, between-group study was conducted to test our hypotheses (see Figure 1 for design details). Ethical approval was gained from the Ethics Committee of the Faculty of Behavioural and Social Sciences at the University of Groningen, Netherlands, and participants signed an informed consent form before the study began. After signing the consent form, participants were given unique study identification codes. Identities of the participants were kept confidential. Next, all participants received an e-mail from the researcher with an electronic link to the instructions and questionnaires. The first set of questionnaires served as a baseline measurement and consisted of the demographic questionnaire, the PANAS, MAAS, PSS, AD ACL, and the MARS. Participants were instructed to complete all the questionnaires at the day of their yoga practice, with at least two hours after the yoga practice in order to avoid acute effects of the practice on outcome measures and not more than 6 hours after the yoga practice.

One week after the baseline measurements, participants in the TY group increased their ashtanga yoga practice frequency to five times per week for the following two weeks. All TY participants participated in ten 90-minute long ashtanga yoga morning classes, Monday to Friday (07:00 h–08:30 h) for two weeks; these classes were instructed similarly as the classes they had taken previously in the evening time at their regular yoga school.

TY participants completed the PANAS, MAAS, PSS, and AD ACL at the end of the two weeks of the TY practice (the 2-week point). WY participants completed the same questionnaires during the same week as the TY. All participants completed the 2-week-point questionnaire with at least two hours passing after their yoga practice, but not more than 6 hours. WY participants maintained their evening practice (18:00 h–19:30 h).

We utilized ashtanga-style yoga in this study. Ashtanga yoga is considered a vigorous practice as it is characterized by a combination of physically challenging asanas, practiced as appropriate for each individual practitioner, while attempting to maintain mental focus and awareness to the breath, altogether meant to facilitate a meditative movement practice [26]. In the present study, each 90-minute class followed a similar format: check-in, breath awareness and centering, sun salutations, standing poses, sitting poses, back bends, finishing poses, and relaxation. To accommodate different ranges of physical capabilities, variations to each pose were given and participants were encouraged to consistently monitor sensations in body and mind and practice in a noncompetitive and sensible manner.

### 2.4. Data Analysis

For the manipulation check we used an independent samples *t*-test to check for differences in yoga class perception and teacher rating between WY and TY participants at week 1 and also at week 2 of the TY intervention. An independent *t*-test was used to examine differences in baseline scores in age, gender, yoga practice experience, PA, NA, MAAS, PSS, AD ACL, and MARS measures between WY and TY participants. We also used independent sample *t*-tests to examine differences in these variables between WY and TY participants at two weeks. Then, to test the effectiveness of a two-week TY practice in previous WY practitioners, we conducted repeated measures ANOVAs with one related factor on two levels (time: baseline and two-week scores for the dependent variables: PA, NA, MAAS, PSS, and AD ACL scores) and for one unrelated factor (the independent variable: condition, WY and TY). Additionally, we conducted a paired *t*-test to investigate for differences in PA, NA, MAAS, PSS, and AD ACL measures from baseline to the 2-week point in WY and TY participants.

Furthermore, to investigate the relationships between PA, NA, MAAS, PSS, and AD ACL scores of all participants at the 2-week point, Pearson's coefficient, *r*, was calculated. Herein, *r* was considered weak (0.10). All values were expressed as mean (M) ± SD, unless otherwise noted. Alpha was set at 0.05 for all statistical analysis and SPSS 20 software was used for all statistical calculations.

## 3. Results

There were no differences in age, gender distribution, and duration of yoga practice experience between groups (Table 1). Furthermore, WY and TY participants utilized similar affect regulatory strategies and no differences in any of the affect regulatory strategies measured were observed (Table 1).

Adherence to the yoga classes in both WY and TY participants was outstanding; all 24 participants completed the study. Only one TY participant missed one class (third day of the TY practice) due to a headache, yet this participant was included in all analyses. The manipulation check revealed that the ashtanga yoga classes and the performance of the teacher were perceived similarly by the TY and WY participants during the 2-week duration of the study (Table 2).

### 3.1. Positive and Negative Affect

**Figure 2:**
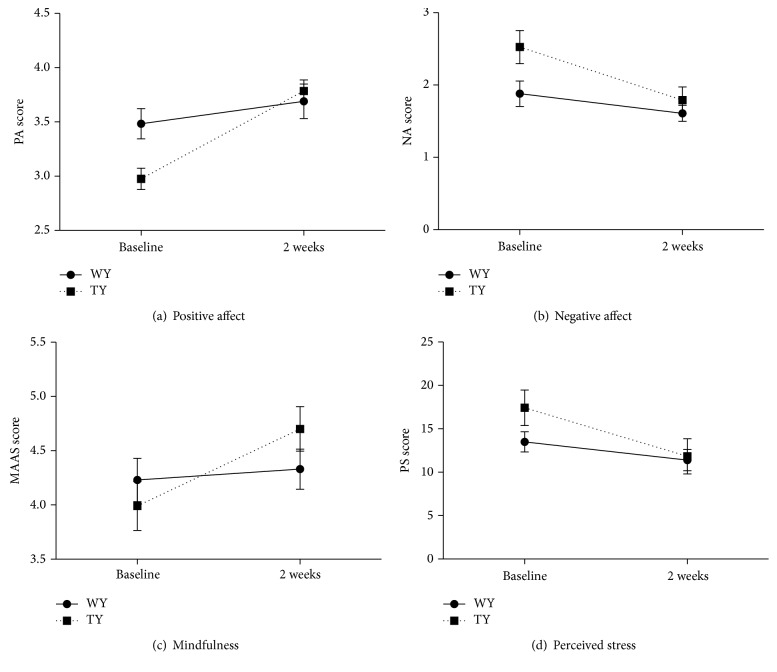
Positive affect (a) and negative affect (b) scores as measured by the PANAS, mindfulness scores (measured by MAAS), and perceived stress (measured by PSS) in yoga practitioners practicing under a Western approach to practice (WY) and practitioners with an existing WY practice who underwent the two-week traditional practice approach (TY) at baseline (0 weeks) and at 2-week-point TY practice (2 weeks). Data are represented as mean ± SD.

Significant baseline differences were apparent between both groups in PA (*p* = 0.007) and NA (*p* = 0.033) subscales of the PANAS. However, at the 2-week point, no such differences were observed and both groups displayed similar values for PA (*p* = 0.663) and NA (*p* = 0.480). The repeated measures ANOVA for PA showed an effect of time (*F*(1,22) = 26.8, *p* = 0.0001, Partial Eta Squared = 0.549, and observed power = 0.999) and an interaction of time and condition (*F*(1,22) = 9.3, *p* = 0.006, Partial Eta Squared = 0.295, and observed power = 0.827, Figure 2(a)). Paired sample *t*-test revealed that PA scores remained constant in WY participants from baseline to the 2-week point (M = 3.48, SD = 0.48 and M = 3.70, SD = 0.55 at baseline and the 2-week point, resp., *p* = 0.160) while TY practitioners displayed an approximate 27% increase in PA scores from baseline to the 2-week point (M = 2.98, SD = 0.34 and M = 3.78, SD = 0.38 at baseline and the 2-week point, resp., *p* = 0.0001).

Moreover, the repeated measures ANOVA for NA revealed an effect of time (*F*(1,22) = 15.0, *p* = 0.001, Partial Eta Squared = 0.406, and observed power = 9.59) and a trend for a significant interaction between time and condition (*F*(1,22) = 3.1, *p* = 0.092, Partial Eta Squared = 0.124, and observed power = 0.392, Figure 2(b)). Notably, the paired sample *t*-test demonstrated an approximate 29% decrease in NA scores in TY participants from baseline to the 2-week point (M = 2.53, SD = 0.76 and M = 1.80, SD = 0.79 at baseline and the 2-week point, resp., *p* = 0.003). As expected, WY participants maintained the same level of NA from baseline to the 2-week point (M = 1.88, SD = 0.61 and M = 1.61, SD = 0.38 at baseline and the 2-week point, *p* = 0.136).

### 3.2. Mindfulness

Both groups displayed similar scores for mindfulness at baseline (*p* = 0.439). A time effect (*F*(1,22) = 9.4, *p* = 0.006, Partial Eta Squared = 0.299, and observed power = 0.83) and interaction between time and condition (*F*(1,22) = 5.3, *p* = 0.031, Partial Eta Squared = 0.195, and observed power = 0.59) were observed upon repeated measures ANOVA (Figure 2(c)). Although no difference in mindfulness between WY participants was observed at the 2-week point (*p* = 0.200), TY participants displayed a significant increase (by approximately 17.5%) in their mindfulness scores from baseline (M = 4.00, SD = 0.78) to the 2-week point (M = 4.70, SD = 0.64, *p* = 0.006). By contrast, no changes in mindfulness were observed in WY participants from baseline (M = 4.23, SD = 0.54) to the 2-week point (M = 4.33, SD = 0.71, *p* = 0.550). Altogether, these findings indicate that a two-week TY practice is effective in improving one's ability to be present in the moment.

### 3.3. Perceived Stress

While scores for perceived stress tended to be higher at baseline (by approximately 22%), albeit nonsignificantly (*p* = 0.131) in TY (M = 17.42, SD = 7.08) compared to WY (M = 13.50, SD = 3.69) participants, both groups displayed similar values at the 2-week point (TY: M = 11.83, SD = 7.04; WY: M = 11.40, SD = 3.86, *p* = 0.864). Paired sample *t*-test analysis demonstrated a trend towards a significant decrease in perceived stress in TY participants from baseline to the 2-week point (*p* = 0.063, Figure 2(d)). This is indicative of a beneficial effect of TY on perceived stress. Repeated measures ANOVA revealed no effect of time (baseline, two-week point) nor an interaction of time and condition.

### 3.4. Arousal States

Table 3 displays scores for arousal states at baseline and the 2-week point. At baseline TY participants displayed a significantly higher EA value compared to WY participants. This showed as approximately 28% higher energy score in TY compared to WY participants, while tiredness scores were similar between both groups at baseline (Table 3). Repeated measures ANOVA for EA revealed a significant effect of time (*F*(1,22) = 4.18, *p* = 0.048, Partial Eta Squared = 0.181, and observed power = 0.516) and a significant interaction between time and condition (*F*(1,22) = 7.303, *p* = 0.014, Partial Eta Squared = 0.267, and observed power = .729). While no differences were observed between both groups at the 2-week point, paired sample *t*-test showed a ≈15% decrease in EA scores in TY participants from baseline to the 2-week point (*p* = 0.004). This showed as a decrease in energy scores (*p* = 0.005), as scores for tiredness remained unchanged in this period for TY (*p* = 0.344). EA scores for WY participants remained similar from baseline to the 2-week point (*p* = 0.702).

Scores for TA, as well as for its subscales tension and calmness, were all similar for both groups at baseline (Table 3). No effect of time or interaction between condition and time was observed by repeated measures ANOVA for TA or its subcomponents tension and calmness. Also paired *t*-test did not reveal any change in any of these parameters from baseline to the 2-week point in WY or TY participants.

### 3.5. Correlations between PA, NA, MAAS, PSS, and AD ACL Scores of All Participants at the 2-Week Point

Several relationships across parameters of emotional well-being were observed at the 2-week point (*n* = 24). A moderate positive relationship was found between PA and mindfulness (*r* = 0.436, *p* = 0.017) indicating that as participants felt more positive, they also experienced more mindfulness. Moreover, strong positive relationships were observed between NA and EA (*r* = 0.502, *p* = 0.015) and NA and perceived stress (*r* = 0.633, *p* = 0.000), as well as a moderate positive relationship between EA and perceived stress (*r* = 0.383, *p* = 0.087). All in all these positive relationships suggest that as participants perceived more stress and energetic arousal, they concurrently felt more negative.

Additionally, a strong negative relationship was observed between PA and perceived stress (*r* = −0.503, *p* = 0.017), illustrating that as participants felt more positive they perceived less stress. Further, strong negative relationships were reported between mindfulness and NA (*r* = −0.483, *p* = 0.017) as well as mindfulness and perceived stress (*r* = −0.692, *p* = 0.000). Moderate negative relationships were found between PA and EA (*r* = −0.349, *p* = 0.103) and NA and TA (*r* = −0.362, *p* = 0.089). In sum, these negative relationships imply that the more mindful the participants were, the less stressed and negative they felt.

## 4. Discussion

While the benefits of yoga interventions for emotional well-being have been widely explored in previously yoga-naïve participants practicing under a more recreational, Western approach (i.e., 1–3 times weekly [11, 13–15, 17]), the effects of an ongoing and more traditional daily practice approach have so far only been substantiated by cross-sectional survey study with a large dropout rate [21]. We aimed to directly evaluate the effectiveness of a short-term (2-week) traditional yoga practice approach (TY) with an ongoing Western yoga practice approach (WY) on parameters of emotional well-being. Results of the present study demonstrate that several parameters of emotional well-being were improved in TY participants at the completion of the WY intervention suggesting clear benefits of a TY compared to maintaining WY. More precisely, completing a short-term, 2-week, five-times-per-week morning practice in participants who previously practiced yoga in the evenings once or twice a week led to significant improvements in their scores for PA, NA, and mindfulness, alongside a trend for improved levels of perceived stress. Altogether, our results inspire the utilization of a yoga practice approach that is based on careful consideration of the frequency and time of the day to elicit the best benefits for emotional well-being.

In terms of a particular yoga practice approach (frequency and time of practice) and its efficiency in benefiting parameters of emotional well-being, our results are difficult to compare to previous research, as the effects of different yoga practice approaches have not been previously explored directly. Yet, our yoga intervention results undermine Ross et al. [21] findings from their American cross-sectional survey in Iyengar yoga practitioners. The authors of the previous study observed that practice frequency was an independent predictor of subjective well-being and mindfulness, while it was also negatively related to sleep disturbance. Importantly, subjective well-being and sleep quality were further improved by each day of home practice herein.

Moreover, our findings substantiate findings from other exercise studies. In addition, anecdotal observation holds, and well-known yoga teachers recognize, that a committed yoga practice (most days of the week) allows for the positive effects of yoga on parameters of mental and emotional well-being to manifest (Yoga Sutra 1.14 [27]). Furthermore, previous exercise studies highlighted that it is a regular, frequent practice that is associated with mental health benefits, facilitating the prevention of depression and anxiety [28, 29]. For example, Knab et al. found that low reported exercise frequency was significantly associated with several measures of psychopathology (including depression and anxiety) while perceived stress was significantly lowered as reported exercise frequency increased [29]. Altogether, exercise studies underline that a regular and consistent exercise paradigm has significant positive impact on emotional health.

Arguably, it may be that either frequency of practice or switching to a morning practice alone may bring about these favorable changes observed in the present study. From empirical exercise studies, we presuppose that consistency in practice approach, and thereby in frequency and time of practice, is a crucial factor herein. Notably, a recent animal study on scheduled physical activity (in mice) suggests that timing of exercise could help shift physiological rhythms to realign better with external environment [30]. Authors also suggested that regularly scheduled physical activity could potentially delay, or even prevent, development of disease. Herein, a consistent early morning yoga practice may favorably affect physiopsychological parameters, such as the ones measured in our study and dynamics required to better face the challenges and stressors of daily life.

It is worth noting that we observed baseline differences in PA, NA, and EA between WY and TY participants. Participation in either TY or WY was not randomly assigned; participants were invited to join a group to either maintain their regular WY practice or join the TY for two weeks in order to increase their yoga practice up to 5 times a week. It is possible that TY participants, who as a group initially presented with lower PA and higher NA and EA, were intrinsically more attracted to participate in such a TY intervention. Importantly, such baseline differences appear not to be due to different affect regulatory patterns as both groups reported using similar affect regulatory strategies at baseline. Due to resource limits it was not possible to carry out a randomized controlled study or a crossover design and let WY participants engage in TY. This is a clear limitation for the present study and should be taken into account for the next step.

Nevertheless, our findings are promising as they indicate that healthy individuals presenting with lower PA and higher NA clearly benefit from an even short-term TY intervention. Previous studies already highlighted that introducing middle-aged yoga-naïve participants with symptoms of high distress and anxiety to 1–3-times-per-week yoga led to improved outcomes of emotional well-being including an improvement in affect, mood, perceived stress, anxiety and depression, and mindfulness [13, 14, 17, 31, 32]. In light of our findings, it is intriguing to ponder whether these outcome measures could further improve by increasing the frequency towards daily yoga practice and/or switching to a morning practice. Future research on this area is warranted.

Moreover, TY practitioners initially presented with higher energy scores leading to an increased EA score compared to WY participants at baseline. Unexpectedly, energy scores (as well as EA scores) significantly decreased, reaching similar levels—as displayed by WY participants at 2 weeks. In light of the observed benefits of TY on affect, mindfulness, and perceived stress and the observed strong positive relationship between EA and NA as well as the moderately strong negative relationship between EA and PA (Table 3), this decrease in energy and EA scores appears, at first sight, puzzling. However, taking into account the short-term nature of this intervention (two weeks), it is conceivable that for the TY participants in whom there was the switch from evening to early morning practice and increasing the frequency of the practice, the decreased energy scores undermine an ongoing process of adaptation to new (practice) conditions. It has to be remembered that ashtanga yoga is a physically demanding practice [26]. Although TY participants were encouraged to engage in each practice with awareness to how the body feels and options for adapting the practice were continuously given, increasing the practice frequency from one to five days per week is an exercise increase that requires adaptation. Moreover, participants shifted from an evening to an early morning practice (07:00 h–08:30 h), requiring reorganization of daily schedules. It may well be that more time is required to adapt to such a new routine.

Yoga is a contemplative movement practice, also referred to as mindfulness in motion: it is constituted by inward concentration and awareness of breath and movement while simultaneously monitoring sensations on a physical, mental, and emotional level. Mindfulness is commonly assessed by the MAAS and MAAS scores have established relationships with parameters of emotional well-being [18, 23, 33]. Our data support this as we also observed strong positive relationships between mindfulness scores (obtained by MAAS) and various aspects of well-being, namely, PA, NA, and perceived stress. It would be valuable to follow whether the observed favorable changes in mindfulness, PA, NA, and perceived stress obtained by the 2-week TY intervention would increase by a long-term TY practice and, also, if the changes would be maintained after returning to a WY practice.

## 5. Conclusion

The present study demonstrates that short-term, 2-week TY compared to ongoing WY led to improved emotional well-being as evidenced by increased PA and mindfulness and decreased NA as well as a trend for decreased perceived stress. Given the increased popularity of yoga in the Western world alongside the already beneficial effects of WY on emotional well-being, our findings lend practical application and promising support to yoga as an evidence-based practice among healthy yoga practitioners.

## Figures and Tables

**Table 1:** Baseline demographics and affect regulation style.

	WY (*n* = 12)	TY (*n* = 12)	*t*	*p *
Demographics				
Age, mean ± SD	30.2 ± 7.9	30.9 ± 8.3	−0.823	0.823
Gender, *n* (%) female	9 (75.0)	11 (91.7)	−1.076	0.294
Yoga practice experience				
Overall mean ± SD	1.7 ± 1.3	2.00 ± 1.4	−0.616	0.544
*n* (%)				
2–6 months	2 (16.7)	1 (8.3)		
Up to 1 year	4 (33.3)	4 (33.4)		
Up to 2 years	4 (33.3)	3 (25.0)		
<2 years	2 (16.7)	4 (33.3)		
Affect regulation style,				
mean ± SD				
Active distraction	3.16 ± 0.89	3.04 ± 0.79	−0.339	0.739
Cognitive engagement	3.38 ± 1.42	3.63 ± 0.73	0.540	0.594
Behavioral engagement	3.14 ± 0.98	3.10 ± 0.64	−0.123	0.903
Venting/expressing affect	3.22 ± 1.20	3.43 ± 0.90	0.500	0.622
Passive distraction	1.85 ± 0.25	2.19 ± 0.25	1.080	0.292
Rumination/withdrawal	3.38 ± 0.25	3.04 ± 0.25	0.121	0.383
Waiting/reframing	2.27 ± 0.28	2.77 ± 0.28	0.055	0.957

Baseline demographic characteristics and affect regulation style (Measure of Affect Regulatory Style Score, MARS) in yoga practitioners practicing under a Western approach to practice (WY) and practitioners with an existing WY practice who were about to start the 2-week traditional practice approach (TY). Data are expressed in mean ± SD unless otherwise specified. Independent Student's *t*-test was performed to compare means between groups. *t* = *t*-value, *p* = *p* value.

**Table 2:** Rating of yoga classes and yoga teacher during the intervention.

	WY (*n* = 12)	TY (*n* = 12)	*t*	*p *
Rating of yoga class	8.1 ± 1.2	7.9 ± 1.5	−2.943	0.794
Rating of yoga teacher	8.5 ± 1.1	8.9 ± 0.9	0.837	0.234

Overall rating of yoga classes (“How do you feel about class today?”) and yoga teacher performance (“How do you rate the performance of the yoga teacher today?”) presented in mean ± SD. For yoga practitioners maintaining their Western approach to practice (WY) the average of the two yoga classes that were attended in this two-week period was calculated. For yoga practitioners who underwent the 2-week traditional yoga practice approach (TY), averages of ratings obtained after each of the 10 classes were obtained. Independent Student's *t*-test was performed to compare means between groups. *t* = *t*-value, *p* = *p* value.

**Table 3:** Arousal states.

	WY	TY	*t*	*p *
Energetic arousal				
Baseline	23.18 ± 5.04	27.33 ± 2.02	2.643	0.015
2-week point	23.18 ± 3.70	23.33 ± 2.87	0.112	0.912
Tense arousal				
Baseline	26.45 ± 2.77	27.08 ± 4.08	0.523	0.673
2-week point	25.82 ± 3.52	26.25 ± 2.34	0.354	0.730
Energy scores				
Baseline	10.27 ± 4.26	14.25 ± 2.52	2.750	0.012
2-week point	11.00 ± 3.52	12.17 ± 3.60	−1.071	0.298
Tiredness scores				
Baseline	12.90 ± 2.34	13.08 ± 1.88	0.189	0.845
2-week point	12.18 ± 2.13	13.92 ± 1.83	2.091	0.298
Tension scores				
Baseline	16.55 ± 2.54	14.92 ± 5.10	−0.963	0.350
2-week point	16.36 ± 3.67	16.00 ± 3.13	−0.262	0.800
Calmness scores				
Baseline	9.91 ± 2.21	12.17 ± 3.61	1.791	0.089
2-week point	9.45 ± 2.01	10.25 ± 2.45	0.843	0.408

Energetic arousal, tense arousal, energy, tiredness, tension, and calmness scores as measured by the AD ACL in yoga practitioners practicing under a Western approach to practice (WY) and practitioners with an existing WY practice who underwent the 2-week traditional practice approach (TY) at baseline (0 weeks) and at the end of the 2-week TY practice (2 weeks). Data are represented as mean ± SD. *t* = *t*-value, *p* = *p* value.
